# Pharmacokinetics of Doramectin After Subcutaneous and Intramuscular Administrations in Siamese Freshwater Crocodiles (*Crocodylus siamensis*)

**DOI:** 10.3390/ani16203289

**Published:** 2026-10-19

**Authors:** Arin Leksansern, Saranya Poapolathep, Narumol Klangkaew, Napasorn Phaochoosak, Kraisiri Khidkhan, Mario Giorgi, Marcos Pérez-López, Orhan Corum, Kamil Uney, Elena Badillo, Pedro Marín, Amnart Poapolathep

**Affiliations:** 1 Department of Pharmacology, Faculty of Veterinary Medicine, Kasetsart University, Bangkok 10900, Thailand; arin.l@ku.th (A.L.); saranya.po@ku.th (S.P.); fvetnak@ku.ac.th (N.K.); anocha.p@ku.th (N.P.); kraisiri.kh@ku.th (K.K.); 2 Department of Veterinary Sciences, University of Pisa, 56124 Pisa, Italy; mario.giorgi@unipi.it; 3 Faculty of Veterinary Medicine, University of Extremadura, 10003 Caceres, Spain; marcospl@unex.es; 4 Department of Pharmacology and Toxicology, Faculty of Veterinary Medicine, University of Hatay Mustafa Kemal, Hatay 31060, Türkiye; orhancorum46@hotmail.com; 5 Department of Pharmacology and Toxicology, Faculty of Veterinary Medicine, University of Selcuk, Konya 42130, Türkiye; kuney@selcuk.edu.tr; 6 Department of Pharmacology, Faculty of Veterinary Medicine, University of Murcia, 30100 Murcia, Spain; ebp2@um.es (E.B.); pmarin@um.es (P.M.)

**Keywords:** doramectin, pharmacokinetics, Siamese crocodiles, dosage regimen, HPLC-FLD

## Abstract

Doramectin (DRM) is a broad-spectrum macrocyclic lactone antiparasitic agent widely used in veterinary medicine. Its pharmacokinetics in reptiles, particularly Siamese freshwater crocodiles (*Crocodylus siamensis*), remain limited. This study aimed to characterize the pharmacokinetic profiles of DRM following subcutaneous (SC) and intramuscular (IM) administration in Siamese crocodiles. Twenty clinically healthy male crocodiles were randomly allocated into four groups (*n* = 5 per group) and administered a single dose of DRM at 0.2 or 0.3 mg/kg body weight (b.w.) via either the SC or IM route. Blood samples were collected from pre-dose to 60 days after administration, and plasma DRM concentrations were quantified using a validated high-performance liquid chromatography method with fluorescence detector. Pharmacokinetic parameters were determined by non-compartmental analysis. Plasma DRM concentrations remained quantifiable through day 60 in 19 of 20 animals. Geometric mean maximum plasma concentrations (C_max_) were 14.73 and 20.28 ng/mL following SC administration at 0.2 and 0.3 mg/kg, respectively, and 24.48 and 36.34 ng/mL following IM administration. Significantly higher C_max_ values were observed at 0.3 mg/kg for both routes (*p* < 0.05). Median T_max_ values were 11 and 9 days following SC administration and 3 and 4 days following IM administration at 0.2 and 0.3 mg/kg, respectively. The geometric mean terminal elimination half-lives (T_1/2λz_) were 5.37 and 9.73 days for SC administration and 7.38 and 9.97 days for IM administration at 0.2 and 0.3 mg/kg, respectively. Systemic exposure increased with dose. The geometric mean AUC_0–last_ increased numerically from 368.84 to 491.87 ng × day/mL following SC administration and significantly from 372.45 to 643.97 ng × day/mL following IM administration (*p* < 0.05). Intramuscular administration resulted in higher C_max_ and more rapid attainment of peak plasma concentrations than SC administration. These findings demonstrate prolonged systemic persistence and dose-related pharmacokinetic changes in DRM in freshwater crocodiles and provide species-specific information to support further evaluation of dosage regimens.

## Simple Summary

Pharmacokinetic information on doramectin (DRM) in reptiles is very limited, and appropriate dosages are often based on information from other animal species. This study investigated how DRM is absorbed and remains in the blood of freshwater crocodiles (*Crocodylus siamensis*) after a single subcutaneous or intramuscular administration at two doses. DRM was detectable in plasma for up to 60 days following both administration routes in most animals. Higher doses resulted in significantly greater maximum plasma concentrations and overall drug exposure, while intramuscular administration resulted in more rapid attainment of peak plasma concentrations than subcutaneous administration. These findings provide species-specific pharmacokinetic information to inform future evaluation of dosing strategies in this species.

## 1. Introduction

The Siamese crocodile (*Crocodylus siamensis*; *C. siamensis*) is an economically important freshwater crocodilian species in Southeast Asia and is also of considerable conservation concern [1]. Historically, the species was widely distributed across Southeast Asia, but its current global distribution has become greatly reduced and fragmented, representing approximately 20% of its historical distribution [2]. It was classified as Critically Endangered by the International Union for Conservation of Nature (IUCN) in 2012 and remains among the least-studied crocodilian species [3]. Despite the substantial decline in wild populations, captive farming of *C. siamensis* has been established and plays an important role in the commercial production of skins, meat, and leather products, as well as in conservation and sustainable utilization of the species [4]. However, maintaining the health and productivity of captive crocodiles remains challenging, particularly because of parasitic infections. Parasitic infections can have both clinical and subclinical effects on crocodile health and production, including impaired growth, reduced production efficiency, increased mortality, and damage to commercially valuable skin, thereby increasing the economic burden associated with parasite control and farm management [5,6,7].

Doramectin (DRM) is a semisynthetic avermectin belonging to the 16-membered macrocyclic lactone class which is widely used as a broad-spectrum antiparasitic agent in veterinary medicine. Developed through chemical modification of ivermectin, a fermentation-derived compound originally produced by *Streptomyces avermitilis*, DRM exhibits potent activity against a wide range of endoparasites and ectoparasites, particularly nematodes and arthropods [8,9]. Its antiparasitic effect is mediated through binding to glutamate-gated and γ-aminobutyric acid (GABA)-gated chloride channels in parasite nerve and muscle cells, resulting in membrane hyperpolarization, paralysis, and eventual parasite death [10]. The safety of avermectins in mammalian hosts is largely attributed to P-glycoprotein mediated efflux (P-gp) at the blood–brain barrier, which limits drug accumulation within the central nervous system and helps maintain concentrations below toxic levels [11]. In addition to its antiparasitic activity, DRM possesses high lipophilicity, which facilitates extensive tissue distribution and prolonged persistence within the host [12,13]. The pharmacokinetics of DRM have been investigated in several mammalian species, including ruminants [14,15,16,17], rabbits [18], dogs [19], and horses [20].

In contrast, information regarding the pharmacokinetics and rational use of DRM in reptiles remains limited. Current reptilian dosing regimens are commonly extrapolated from other animal species [21]. Unlike mammals, reptilian physiological processes are strongly influenced by environmental temperature, which can contribute to variation in drug disposition among individuals [22]. Other physiological factors may also influence drug disposition. For example, the renal portal system, reptilian-type nephrons, feeding status, and metabolic activity may affect drug absorption, distribution, metabolism, and elimination [23,24].

Despite the established use of DRM as a broad-spectrum antiparasitic agent in veterinary medicine, pharmacokinetic information for DRM in *C. siamensis* remains unavailable. Species-specific pharmacokinetic evaluation is therefore necessary to support the rational development of an appropriate dosage regimen for this species. The objectives of the present study were to characterize the pharmacokinetic disposition of DRM following subcutaneous and intramuscular administrations and to establish a pharmacokinetically informed dosage regimen and route of administration of DRM in *C. siamensis*.

## 2. Materials and Methods

### 2.1. Animals

Twenty male Siamese freshwater crocodiles, approximately two years of age, were used in the study. The animals had body weights ranging from 11 to 15.2 kg and body lengths of 121–149 cm, measured from the head to the tip of the tail. All crocodiles were obtained from and housed at Wongveerakit Crocodile Farm, Kanchanaburi Province, Thailand, in two separate cement ponds according to the route of administration. Each pond had an approximate water surface area of 50 m^2^ and a water depth of approximately 25 cm. Both ponds were supplied with water from the same source and maintained under the same husbandry practices. Water temperature ranged from 27 to 34 °C during the study period, and environmental conditions were considered comparable between ponds. The animals were considered clinically healthy based on physical examination and were monitored throughout the study by daily observation of behavior, activity, and general clinical condition. None of the crocodiles had received any drugs or other pharmacologically active substances for at least 2 months before study initiation. The experimental protocol was conducted in accordance with the guidelines for the use of animals and was approved by the Kasetsart University Institutional Animal Care and Use Committee (approval code: ACKU68-VET-018).

### 2.2. Drugs and Chemicals

Doramectin VETRANAL™ analytical standard (96.6% purity; Supelco, Sigma-Aldrich, Buchs, Switzerland) was used as the reference standard, while ivermectin (IVM) pharmaceutical secondary standard, certified reference material (Sigma-Aldrich, Buchs, Switzerland), was used as the internal standard (IS). The commercial DRM formulation, Dectomax^®^ injectable solution containing 10 mg/mL (1%) DRM, was manufactured by Zoetis Inc. (Kalamazoo, MI, USA). Stock solutions of DRM and IVM were prepared in acetonitrile at a concentration of 1 mg/mL. Acetonitrile and chloroform (HPLC grade) were purchased from RCI Labscan Limited (Bangkok, Thailand). 1-Methylimidazole (ReagentPlus^®^, 99%; Sigma-Aldrich, Buchs, Switzerland) and trifluoroacetic anhydride (99+%; Thermo Scientific, Waltham, MA, USA) were used for sample preparation and derivatization.

### 2.3. Experimental Design

Twenty crocodiles were weighed and randomly allocated into four treatment groups (*n* = 5 per group) using a simple randomization procedure without stratification by age or body weight. The study followed a parallel-group design, with each group receiving a single dose of DRM at either 0.2 or 0.3 mg/kg body weight (b.w.) via the subcutaneous (SC) or intramuscular (IM) route. In the absence of species-specific pharmacokinetic data in crocodiles, the selected doses were based on pharmacokinetic studies of DRM in rabbits and goats [18,25]. The inclusion of the IM route was additionally informed by reported pharmacokinetic data in horses [20]. For SC administration, DRM was injected into the cranial dorsal region. For IM administration, DRM was injected into the right biceps brachii muscle of the forelimb using a 22-gauge, 1.5-inch needle. Blood samples (1.5 mL) were collected from the tail vein into heparinized tubes before drug administration (0 h) and at 4, 8, and 10 h, followed by 1, 2, 3, 4, 5, 6, 7, 9, 11, 13, 15, 17, 19, 21, 24, 27, 30, 33, 36, 39, 42, 45, 48, 52, 55, and 60 days after administration. During blood collection, the crocodiles were manually restrained by securing the jaws and controlling the anterior and posterior portions of the body. Following each venipuncture, the puncture site was monitored until bleeding had completely ceased, and the animals were released only after hemostasis was confirmed. Plasma was separated by centrifugation at 1986× *g* for 15 min and stored at −20 °C until analysis.

### 2.4. Analysis of Doramectin Concentration in Plasma

An extraction method for DRM determination using a liquid–liquid extraction procedure was adapted from the method reported by Gokbulut et al. [18]. Plasma samples stored at −20 °C were thawed at room temperature, and an aliquot of 180 µL was transferred to a tube. Ivermectin (IVM) was added as the internal standard (IS) at a concentration of 10 ng/mL in a volume of 20 µL, followed by the addition of 400 µL of acetonitrile. The mixture was vortexed thoroughly, after which 800 µL of chloroform was added. The samples were centrifuged at 4000× *g* for 10 min at 4 °C to facilitate phase separation. The organic phase was collected, and the remaining precipitate was re-extracted with chloroform. The organic phases from both extraction steps were combined and evaporated to dryness under a gentle stream of nitrogen at 45 °C. The dried extracts were reconstituted with 60 µL of a 1:1 (*v*/*v*) mixture of 1-methylimidazole and acetonitrile. Derivatization was subsequently performed by adding 90 µL of a 1:2 (*v*/*v*) mixture of trifluoroacetic anhydride and acetonitrile. The derivatized samples were filtered through a 0.22 µm PVDF syringe filter and transferred to autosampler vials for HPLC analysis.

Chromatographic separation was performed using an Agilent 1260 series HPLC system (Agilent Technologies, Santa Clara, CA, USA) equipped with dual pumps, an autosampler, and column temperature control. Separation was achieved using a UMISIL™ C18 analytical column (5 µm particle size, 150 × 4.6 mm i.d.; Thermo Fisher Scientific, Vilnius, Lithuania) with a ZORBAX C18 guard column (4.6 × 12.5 mm, 5 µm; Agilent Technologies, Santa Clara, CA, USA). The column temperature was maintained at 30 °C. The mobile phase consisted of acetonitrile, methanol, and 0.2% acetic acid in Milli-Q water at a ratio of 65:30:5 (*v*/*v*) and was delivered by isocratic condition at a flow rate of 1.1 mL/min. Fluorescence detection was performed at an excitation wavelength of 365 nm and an emission wavelength of 470 nm.

### 2.5. Analytical Method Validation

The chromatographic method was evaluated with reference to the applicable principles of the ICH M10 guideline for bioanalytical method validation [26]. Calibration standards were prepared by spiking DRM working standard solutions into blank plasma collected from Siamese crocodiles before drug administration, yielding 11 concentration levels ranging from 0.1 to 50 ng/mL (0.1, 0.25, 0.5, 0.75, 1, 1.25, 2.5, 5, 10, 25, and 50 ng/mL). Quality control (QC) samples were prepared at four concentrations (0.1, 0.25, 10, and 40 ng/mL). Accuracy, precision, and extraction recovery were assessed using five replicates per QC level over three separate days. Selectivity was evaluated using blank plasma from individual *Crocodylus siamensis* and *Crocodylus porosus* by examining chromatograms for interfering signals at the retention times of DRM and the internal standard. Matrix effect was evaluated using low and high QC samples. Carry-over was assessed by injecting blank samples following the upper limit of quantification (ULOQ) calibration standard, with acceptance criteria of an analyte response ≤20% of the response at the lower limit of quantification (LLOQ) and an IS response ≤5% of the IS response at the LLOQ. Bench-top stability was assessed at room temperature for 4 h, and freeze–thaw stability was evaluated over three cycles. Processed-sample stability was assessed in the autosampler at room temperature under routine analytical conditions. Reinjection reproducibility was not evaluated because no processed study samples were reinjected during sample analysis. Dilution integrity was not evaluated because no study samples required dilution. Formal long-term storage stability at −20 °C was not assessed.

### 2.6. Pharmacokinetic Analysis

Pharmacokinetic analysis of DRM was performed using PKanalix 2023 (PKanalix^TM^ R1; 2023; Lixoft Software, Paris, France). Non-compartmental analysis was applied to the plasma concentration–time data obtained following subcutaneous and intramuscular administration. The pharmacokinetic parameters evaluated included the maximum plasma concentration (C_max_), time to maximum concentration (T_max_), terminal elimination rate constant (λ_z_), terminal elimination half-life (T_1/2λz_), area under the plasma concentration–time curve from time zero to the last measurable concentration (AUC_0–last_), area under the curve extrapolated to infinity (AUC_0–∞_), Percentage of area under the curve extrapolated to infinity (AUC_%extrap_), apparent volume of distribution (V_z_/F), apparent clearance (Cl/F), and mean residence time (MRT). Concentrations below the LLOQ were assigned a value of zero for data handling.

### 2.7. Statistical Analysis

Pharmacokinetic parameters are summarized as geometric means (GM) with minimum and maximum values, except for T_max_, which is summarized as the median with minimum and maximum values and was not subjected to inferential statistical testing. Geometric means were used to summarize pharmacokinetic parameters, consistent with the natural log-transformed scale used for dose-group comparisons. Plasma concentration–time data and pharmacokinetic parameters from the present study used for descriptive interspecies comparisons are presented as arithmetic means ± standard deviations (SDs), calculated from individual-animal data (*n* = 5 per dose group for each treatment group), whereas values from published studies are reported as described in the original publications. Dose groups were compared within each administration route using two-sided Welch’s t-tests on natural log-transformed pharmacokinetic parameters. Levene’s test was used to assess variance homogeneity. A *p* < 0.05 was considered statistically significant.

## 3. Results

Throughout the experimental period, no clinically apparent adverse signs were observed during daily monitoring of behavior, activity and general clinical condition following SC or IM administration of DRM at doses of 0.2 and 0.3 mg/kg. At the final sampling point (day 60), one animal in the SC 0.2 mg/kg group had a plasma DRM concentration below the lower limit of quantification (0.1 ng/mL).

The calibration curves showed good linearity over the concentration range of 0.1–50 ng/mL, with a coefficient of determination (R^2^) of 0.996. The LLOQ was 0.1 ng/mL. Back-calculated calibration standard concentrations met the predefined acceptance criteria of ±20% at the LLOQ and ±15% at the other concentration levels. Accuracy ranged from 87% to 110%, while within-run and between-run precision ranged from 1.1% to 7.9% and 1.2% to 7.4% CV, respectively. Extraction recovery ranged from 76% to 117% across the evaluated QC levels, with consistent recovery observed among replicates within each QC level. Selectivity assessment revealed no interfering signals at the retention times of DRM and the IS in the tested crocodile plasma sources. Matrix effect results met the predefined accuracy criterion of ±15% and precision criterion of ≤15% CV. No detectable carry-over was observed following injection of the ULOQ standard. Bench-top stability at room temperature for 4 h, freeze–thaw stability over three cycles, and processed-sample stability under the tested autosampler conditions were acceptable. Formal long-term storage stability at −20 °C was not evaluated.

**Figure 1:**
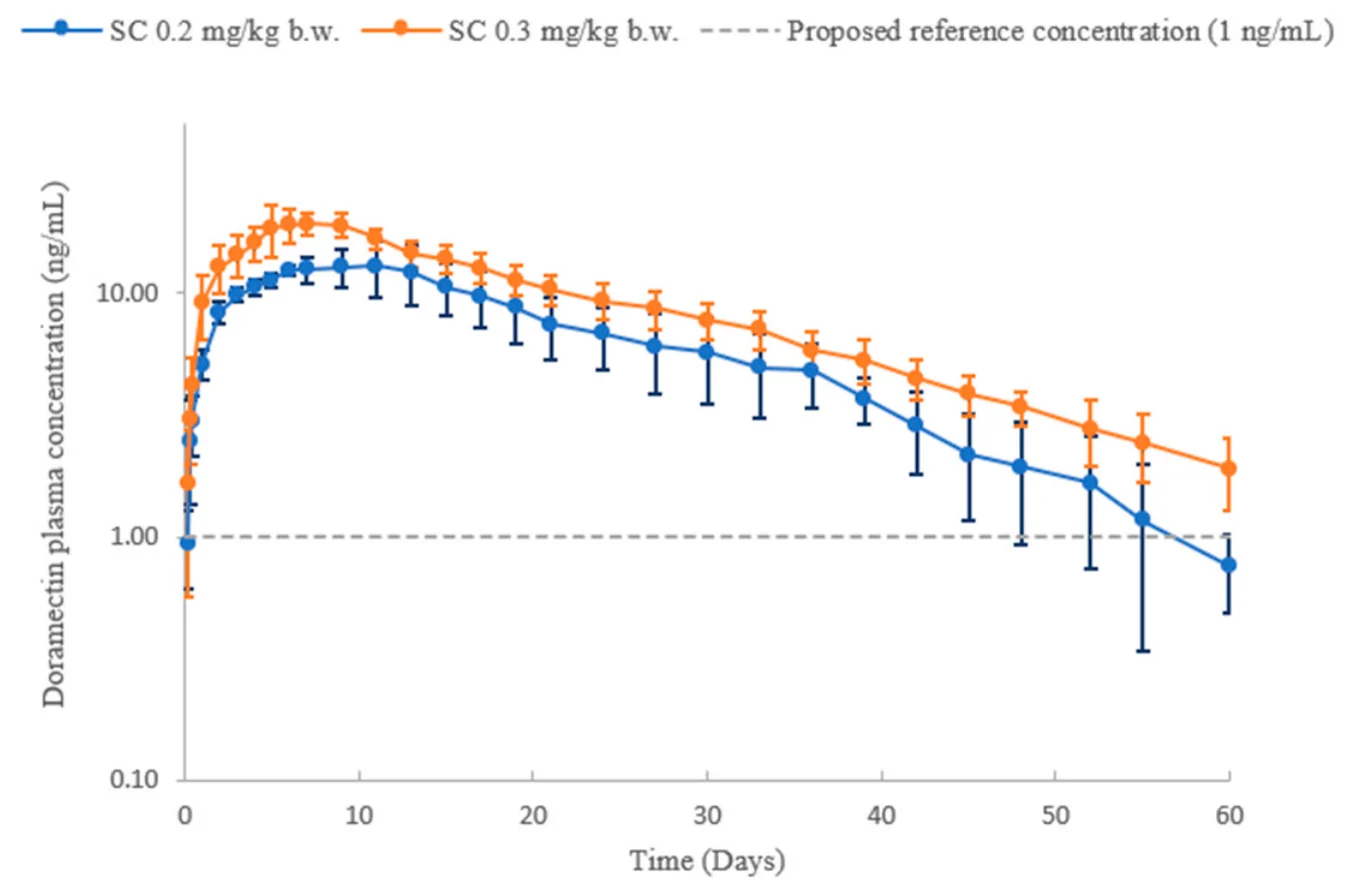
Plasma concentration–time profiles of doramectin following subcutaneous administration at 0.2 and 0.3 mg/kg in freshwater crocodiles (*Crocodylus siamensis*). Data are presented as arithmetic means ± SD (*n* = 5). The dashed line represents the proposed reference concentration of 1 ng/mL [27]. At day 60, one animal in the SC 0.2 mg/kg group had a plasma DRM concentration below the LLOQ (0.1 ng/mL).

**Figure 2:**
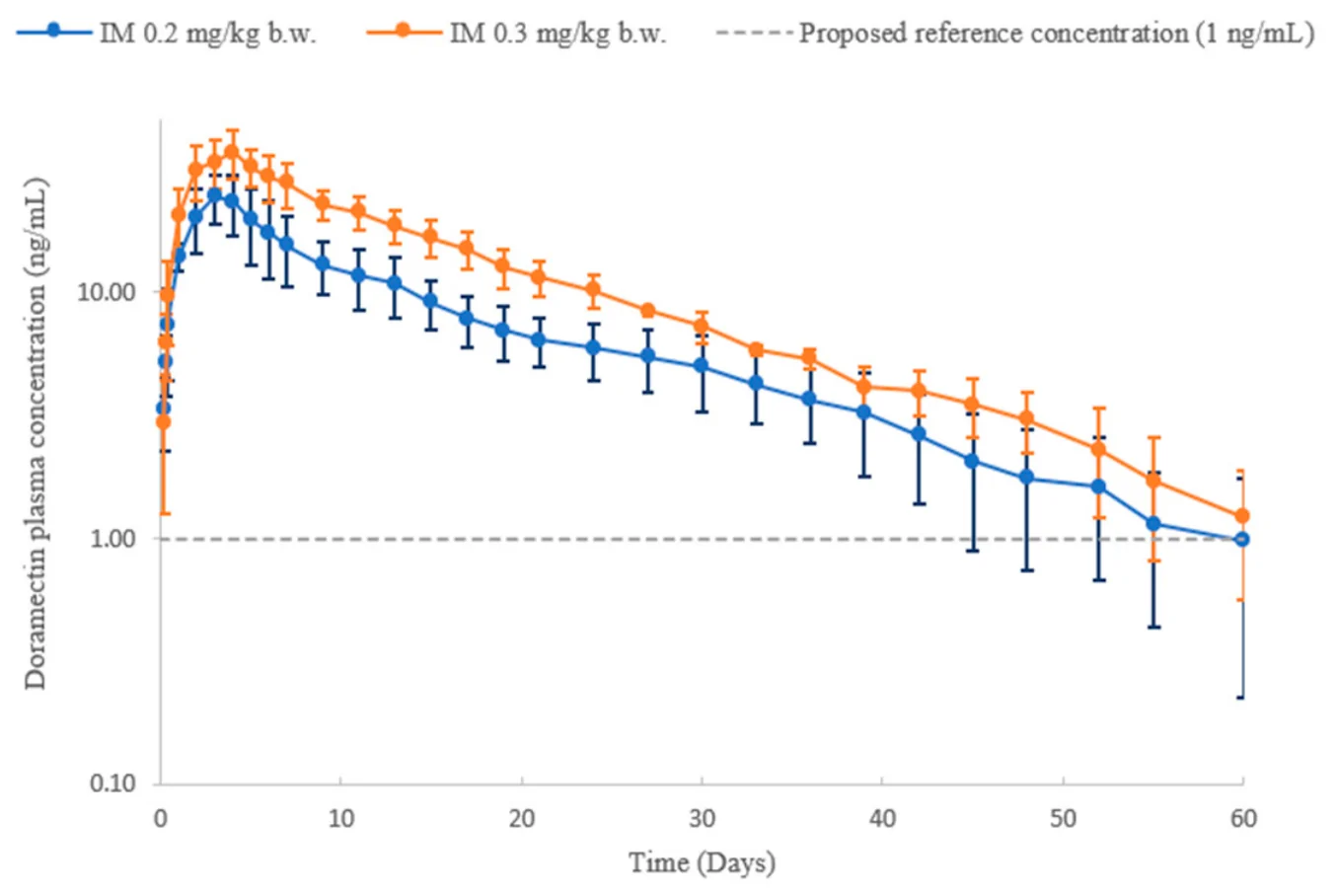
Plasma concentration–time profiles of doramectin following intramuscular administration at 0.2 and 0.3 mg/kg in freshwater crocodiles (*Crocodylus siamensis*). Data are presented as arithmetic means ± SD (*n* = 5). The dashed line represents the proposed reference concentration of 1 ng/mL [27].

DRM remained quantifiable following both administration routes in most animals. The plasma concentration–time profiles showed earlier attainment of peak concentrations following IM administration, while higher doses were associated with greater systemic exposure and prolonged terminal disposition. The corresponding pharmacokinetic parameters are summarized as geometric means with minimum and maximum values, except for T_max_, which is presented as the median with minimum and maximum values. Plasma concentration–time profiles are presented as arithmetic means ± standard deviations at each sampling time (*n* = 5 per dose group), as shown in Figure 1 and Figure 2 for SC and IM administration, respectively. The pharmacokinetic parameters following subcutaneous and intramuscular administration are summarized in Table 1 and Table 2, respectively.

## 4. Discussion

Pharmacokinetic knowledge is important for responsible drug use and the development of appropriate dosage regimens. To our knowledge, no previous pharmacokinetic studies of DRM have been reported in crocodiles. This study provides the first characterization of DRM pharmacokinetics in freshwater crocodiles (*C. siamensis*). The findings offer initial species-specific information on DRM disposition following SC and IM administration and may inform future investigations of dosing strategies in this species. The present study focused on characterizing dose- and route-related pharmacokinetic profiles.

Doramectin showed dose-related changes in its pharmacokinetic profile, with differences between the SC and IM routes. Increasing the dose from 0.2 to 0.3 mg/kg resulted in significantly higher C_max_ values following both SC and IM administration, increasing from 14.73 to 20.28 ng/mL and from 24.48 to 36.34 ng/mL, respectively (*p* < 0.05). Systemic exposure also increased with dose. AUC_0–last_ increased numerically from 368.84 to 491.87 ng × day/mL following SC administration, whereas it increased significantly from 372.45 to 643.97 ng × day/mL following IM administration (*p* < 0.05). AUC_0–∞_ increased from 374.66 to 516.57 ng × day/mL following SC administration and from 378.81 to 660.21 ng × day/mL following IM administration, with significant differences between dose groups for both routes (*p* < 0.05). The geometric mean AUC_%extrap_ was 0.87% and 3.78% following SC administration at 0.2 and 0.3 mg/kg, respectively, and 1.17% and 2.22% following IM administration. Individual values ranged from 0.21% to 7.35% across all groups. In the SC groups, V_z_/F also increased significantly from 4136.90 to 8156.35 mL/kg (*p* < 0.05), whereas V_z_/F was comparable between doses following IM administration. T_1/2λz_ increased from 5.37 to 9.73 days following SC administration and from 7.38 to 9.97 days following IM administration, while MRT increased from 20.18 to 23.03 days in the SC groups and from 17.10 to 17.98 days in the IM groups. IM administration resulted in more rapid attainment of peak plasma concentrations than SC administration at both dose levels, with median T_max_ values of 11 and 9 days following SC administration at 0.2 and 0.3 mg/kg, respectively, and 3 and 4 days following IM administration at 0.2 and 0.3 mg/kg, respectively.

Comparative pharmacokinetic parameters of DRM following SC and IM administration in Siamese crocodiles and other animal species are presented in Table 3. In this table, values from the present study are expressed as arithmetic means ± SD, calculated from individual-animal data, to facilitate comparison with published studies. Values from published studies are reported as described in the original publications. These interspecies comparisons provide a descriptive context for the present findings, and differences in study designs should be considered when interpreting the results. Following SC administration at 0.2 mg/kg, C_max_ in freshwater crocodiles was 14.81 ± 1.70 ng/mL, lower than that reported in sheep (22.75 ± 9.16 ng/mL) [14], goats (16.5 ± 1.2 ng/mL) [25], and calves (32.0 ± 9.3 ng/mL) [15], but higher than that reported in alpacas (6.05 ± 5.34 ng/mL) [16]. At 0.2 mg/kg IM, C_max_ in crocodiles was 25.12 ± 6.27 ng/mL, compared with 33.3 ± 10.5 ng/mL in crossbred horses [20]. At 0.3 mg/kg SC, C_max_ in crocodiles was lower than that reported in rabbits receiving the same dose and route (20.32 ± 1.55 and 34.14 ± 5.47 ng/mL, respectively) [18]. These differences may reflect species-specific physiological and absorption characteristics [28].

A more consistent difference was observed in T_max_. At 0.2 mg/kg SC, crocodiles reached peak plasma concentrations at 10.00 ± 2.24 days, later than sheep (3.0 ± 1.0 days) [14], goats (1.37 ± 0.11 days) [25], calves (3.86 ± 1.77 days) [15], and alpacas (3.83 ± 2.48 days) [16]. At 0.3 mg/kg SC, T_max_ in crocodiles was 7.80 ± 1.64 days, also later than that reported in rabbits (3.10 ± 1.24 days) [18]. In contrast, T_max_ following IM administration at 0.2 mg/kg in crocodiles was 3.40 ± 0.55 days, compared with 2.9 ± 0.9 days in crossbred horses [20]. Overall, the T_max_ profile indicated a route-dependent difference in the rate of drug absorption, with peak plasma concentrations being attained more rapidly following IM administration than SC administration.

Systemic exposure also differed among the compared species. At 0.2 mg/kg SC, AUC_0–last_ in crocodiles was 377.41 ± 85.18 ng × day/mL, higher than that reported in sheep (146.9 ± 25.5 ng × day/mL) [14] and goats (102.3 ± 7.0 ng × day/mL) [25], but lower than that reported in calves (391 ± 110 ng × day/mL) [15]. At 0.3 mg/kg SC, AUC_0–last_ in crocodiles was 492.93 ± 36.43 ng × day/mL, higher than that reported in rabbits (258.40 ± 62.05 ng × day/mL) receiving the same dose and route [18]. Following IM administration at 0.2 mg/kg, AUC_0–last_ in crocodiles was 389.10 ± 124.71 ng × day/mL, compared with 393.6 ± 66.6 ng × day/mL in crossbred horses [20].

The value of T_1/2λz_ also differed among species. At 0.2 mg/kg SC, crocodiles had a T_1/2λz_ of 6.18 ± 4.13 days, longer than that reported in sheep (3.43 ± 1.22 days) [14] and goats (2.6 ± 0.2 days) [25], but shorter than that reported in alpacas (6.20 ± 4.90 days) [16]. Following SC administration at 0.3 mg/kg, the T_1/2λz_ increased to 10.58 ± 4.35 days, exceeding that reported in rabbits receiving the same dose and route (4.48 ± 1.36 days) [18]. At 0.2 mg/kg IM, the T_1/2λz_ in Siamese crocodiles was 8.17 ± 3.71 days, compared with 9.3 ± 4.3 days in crossbred horses [20]. These findings indicate prolonged terminal persistence in crocodiles compared with several of the species included in Table 3, although longer half-lives were reported in alpacas and horses. Prolonged T_1/2λz_ was accompanied by relatively low Cl/F values in crocodiles, which were 549.22 ± 155.11 mL/day/kg in the SC group and 554.33 ± 195.57 mL/day/kg in the IM group, compared with 1400 ± 240 mL/day/kg reported in sheep [14]. These findings suggest that lower apparent clearance may contribute to the prolonged terminal disposition observed in crocodiles. The lower apparent clearance may be consistent with the slower metabolic processes described in reptiles [29].

Direct PK data for DRM at 0.3 mg/kg IM in other species were limited, precluding direct interspecies comparison. This dose was included based on the established 300 μg/kg IM dosage of DRM in swine [30].

Published pharmacokinetic data for macrocyclic lactones in reptiles remain limited compared with those available in domestic mammals. Ivermectin has been used in reptile medicine, particularly in snakes and lizards, with dosing recommendations based largely on clinical experience rather than species-specific pharmacokinetic studies [21,31]. Marked differences in ivermectin tolerance have also been reported among reptile species, with chelonians developing paresis at doses as low as 0.025–0.05 mg/kg, whereas corn snakes tolerated doses up to 1.0 mg/kg without apparent adverse effects [32,33,34]. These findings highlight the variability in response to macrocyclic lactones among reptile species and the limitations of extrapolating dosage between species.

Interpretation of the limited reptilian PK data is further complicated by differences in pharmacokinetic behavior among macrocyclic lactone compounds. In cattle, DRM and ivermectin administered subcutaneously at 0.2 mg/kg produced similar C_max_ values, whereas DRM showed a later T_max_ and greater AUC_0–∞_, which were attributed to lower clearance, lower volume of distribution, and possibly higher bioavailability of DRM [35]. Comparative studies in calves also showed higher and more sustained plasma concentrations of DRM than those of abamectin and several ivermectin formulations, resulting in greater AUC and MRT values [15]. Similarly, DRM produced greater AUC and longer terminal persistence than ivermectin following subcutaneous administration in rabbits, despite no significant difference in C_max_ among the three macrocyclic lactones evaluated [18]. These findings show that macrocyclic lactone compounds can differ in plasma disposition even when administered at the same dose and by the same route. Formulation can further affect DRM disposition, as oil-based formulations producing more prolonged plasma concentrations have been associated with sustained antiparasitic efficacy following SC administration in cattle [36]. Therefore, pharmacokinetic and dosing information obtained for ivermectin or other macrocyclic lactones cannot be assumed to apply directly to DRM.

The prolonged persistence of DRM observed in the present study may support sustained drug exposure at tissues where parasites are located. A close relationship between plasma and tissue concentrations of DRM and ivermectin has been reported, suggesting that plasma concentrations may provide an indication of drug exposure at parasite target sites [27]. DRM exert their antiparasitic effects by disrupting pharyngeal pumping and somatic muscle activity in nematodes, although the in vivo drug concentration required to produce these effects has not been established [37]. In addition, a specific minimum effective concentration (MEC) has not been established for the parasites of freshwater crocodiles. Based on pharmacological studies of macrocyclic lactones, plasma concentrations above 1 ng/mL have been proposed as a theoretical reference level for effective antiparasitic activity against some endoparasites [27]. Following this approach, time (T) >1 ng/mL was estimated in the present study as an indirect measure of the persistence of systemic exposure above this reference concentration. Therefore, T > 1 ng/mL should be interpreted as an exploratory exposure metric rather than a validated pharmacodynamic target.

The estimated T > 1 ng/mL values were approximately 56.9 days following SC administration and 59.4 days following IM administration at 0.2 mg/kg. At 0.3 mg/kg, mean plasma concentrations remained above 1 ng/mL through the final sampling point at 60 days following both routes. Accordingly, T > 1 ng/mL in the 0.3 mg/kg groups could only be estimated as ≥60 days, as the time at which concentrations fell below the reference level was not captured within the sampling period. The higher dose therefore produced greater systemic exposure and was associated with a longer estimated duration above the proposed reference level, although the exact magnitude of this increase could not be determined. IM administration resulted in more rapid attainment of peak plasma concentrations. However, the estimated duration above 1 ng/mL was similar between routes at 0.2 mg/kg. At 0.3 mg/kg, the duration could not be directly compared between routes because mean plasma concentrations in both groups remained above the reference level at day 60. Because 1 ng/mL has not been established as a minimum effective concentration for DRM against parasites of *C. siamensis*, these findings indicate prolonged systemic exposure rather than confirmed antiparasitic efficacy.

Beyond systemic drug exposure, the amount of drug reaching the parasite’s site of action may also influence its pharmacological effect. Drug lipophilicity and the permeability of the helminth’s external surface influence the effective drug concentrations reaching the site of action [38]. The somatic neuromuscular system has also been identified as a major site of action of macrocyclic lactones and is associated with resistance to these drugs [39,40]. A study in *Haemonchus contortus* demonstrated concentration- and time-dependent accumulation of ivermectin and moxidectin within adult nematodes, with increased drug exposure associated with greater drug accumulation in the parasites. The study also highlighted the importance of parasite susceptibility in determining the response to increased drug exposure [41]. Collectively, these findings suggest that the pharmacodynamic effects of macrocyclic lactones may depend not only on systemic drug exposure but also on drug availability at the parasite site and parasite susceptibility. These factors provide a pharmacological context for interpreting the dose- and route-dependent differences in plasma DRM exposure observed in the present study and highlight relevant considerations for future evaluation of antiparasitic efficacy in *C. siamensis*.

However, several limitations should be considered. The small sample size (*n* = 5 per group) and inclusion of only two-year-old male crocodiles may limit the characterization of interindividual variability. The absence of an intravenous group precluded direct estimation of absolute bioavailability, requiring clearance and volume of distribution to be reported as apparent parameters (Cl/F and V_z_/F). Although husbandry and water sources were identical between treatment ponds, individual body temperatures were not monitored, which could influence metabolic rate. Furthermore, safety was assessed via daily clinical monitoring without hematological, biochemical, or histopathological evaluations, and T > 1 ng/mL reflects an exploratory threshold rather than a validated minimum effective concentration against crocodilian parasites. Extended sampling is warranted to refine terminal elimination phase estimates. Future studies should involve larger sample sizes of freshwater crocodiles and investigate DRM concentrations in relevant tissues, given its high lipophilicity and potential for extensive tissue distribution. Integrating plasma and tissue exposure data with parasitological efficacy and safety assessments would provide a comprehensive understanding of DRM disposition and support the refinement of species-specific dosage regimens in *C. siamensis*.

## 5. Conclusions

Doramectin showed prolonged systemic persistence in freshwater crocodiles following a single administration, with dose-related increases in systemic exposure and more rapid attainment of peak plasma concentrations following IM administration than SC administration. These findings provide species-specific pharmacokinetic information to support further evaluation of DRM dose and route in *C. siamensis*. Further studies incorporating parasitological efficacy and pharmacokinetic/pharmacodynamic integration are warranted to further evaluate the studied doses and refine the dosing regimen of DRM in Siamese freshwater crocodiles.

## Figures and Tables

**Table 1:** Pharmacokinetic parameters of doramectin (geometric mean) in freshwater crocodiles (*Crocodylus siamensis*) following subcutaneous administration at 0.2 and 0.3 mg/kg b.w.

Parameter	Unit	Subcutaneous (SC)					
		0.2 mg/kg (*n* = 5)			0.3 mg/kg (*n* = 5)		
		GM	Min	Max	GM	Min	Max
C_max_	ng/mL	14.73	12.86	17.34	20.28 *	18.17	22.04
T_max_	day	11	6	11	9	6	9
AUC_0–last_	ng × day/mL	368.84	246.03	483.77	491.87	450.02	546.91
AUC_0–∞_	ng × day/mL	374.66	246.54	504.69	516.57 *	453.51	575.15
AUC_%extrap_	%	0.87	0.21	4.15	3.78	0.77	7.35
λ_z_	1/day	0.13	0.05	0.20	0.0712	0.0429	0.1545
T_1/2λz_	day	5.37	3.54	13.31	9.73	4.49	16.16
V_z_/F	mL/kg	4136.90	2784.49	7607.89	8156.35 *	4280.97	13,561.14
Cl/F	mL/day/kg	533.81	396.28	811.23	580.76	521.61	661.50
MRT_0–∞_	day	20.18	16.35	23.68	23.03	18.93	25.96

Abbreviations: C_max_ = maximum plasma concentration; T_max_ = time to maximum plasma concentration; AUC_0–last_ = area under the plasma concentration–time curve from time 0 to the last quantifiable concentration; AUC_0–∞_ = area under the plasma concentration–time curve from time 0 to infinity; AUC_%extrap_ = percentage of area under the plasma concentration–time curve extrapolated to infinity; λ_z_ = terminal elimination rate constant; T_1/2λz_ = terminal elimination half-life; V_z_/F = apparent volume of distribution during the terminal phase; Cl/F = apparent clearance; MRT_0–∞_ = mean residence time from time 0 to infinity. T_max_ is presented as median. * Significant difference between dose groups within the same administration route (*p* < 0.05).

**Table 2:** Pharmacokinetic parameters of doramectin (geometric mean) in freshwater crocodiles (*Crocodylus siamensis*) following intramuscular administration at 0.2 and 0.3 mg/kg b.w.

Parameter	Unit	Intramuscular (IM)					
		0.2 mg/kg (*n* = 5)			0.3 mg/kg (*n* = 5)		
		GM	Min	Max	GM	Min	Max
C_max_	ng/mL	24.48	17.76	32.80	36.34 *	22.73	46.39
T_max_	day	3	3	4	4	2	5
AUC_0–last_	ng × day/mL	372.45	233.61	534.52	643.97 *	476.64	744.96
AUC_0–∞_	ng × day/mL	378.81	236.11	556.13	660.21 *	492.36	776.72
AUC_%extrap_	%	1.17	0.32	3.89	2.22	1.21	4.09
λ_z_	1/day	0.09	0.06	0.19	0.07	0.05	0.12
T_1/2λz_	day	7.38	3.57	12.43	9.97	6.03	13.29
V_z_/F	mL/kg	5623.46	2884.36	12,440.77	6539.22	3694.68	11,678.81
Cl/F	mL/day/kg	527.98	359.63	847.07	454.40	386.24	609.31
MRT_0–∞_	day	17.10	15.45	19.75	17.98	15.47	20.32

Abbreviations: C_max_ = maximum plasma concentration; T_max_ = time to maximum plasma concentration; AUC_0–last_ = area under the plasma concentration–time curve from time 0 to the last quantifiable concentration; AUC_0–∞_ = area under the plasma concentration–time curve from time 0 to infinity; AUC_%extrap_ = percentage of area under the plasma concentration–time curve extrapolated to infinity; λ_z_ = terminal elimination rate constant; T_1/2λz_ = terminal elimination half-life; V_z_/F = apparent volume of distribution during the terminal phase; Cl/F = apparent clearance; MRT_0–∞_ = mean residence time from time 0 to infinity. T_max_ is presented as median. * Significant difference between dose groups within the same administration route (*p* < 0.05).

**Table 3:** Comparative pharmacokinetic parameters of doramectin following subcutaneous and intramuscular administration in freshwater crocodiles and other animal species.

Species	Route	Dose (mg/kg)	C_max_ (ng/mL)	T_max_ (day)	AUC_0–last_ (ng × day/mL)	T_1/2λz_ (day)	References
Freshwater crocodiles (*Crocodylus siamensis*)	SC	0.2, 0.3	14.81 ± 1.70, 20.32 ± 1.55	10.00 ± 2.24, 7.80 ± 1.64	377.41 ± 85.18, 492.93 ± 36.43	6.18 ± 4.13, 10.58 ± 4.35	Present Study
	IM	0.2, 0.3	25.12 ± 6.27, 37.35 ± 8.85	3.40 ± 0.55, 3.80 ± 1.10	389.10 ± 124.71, 651.38 ± 103.16	8.17 ± 3.71, 10.32 ± 2.75	Present Study
Sheep (*Ovis aries*)	SC	0.2	22.75 ± 9.16	3 ± 1	146.9 ± 25.5	3.43 ± 1.22	[14]
Goat (*Capra hircus*)	SC	0.2	16.5 ± 1.2	1.37 ± 0.11	102.3 ± 7.0	2.6 ± 0.2	[25]
Calves (*Bos taurus*)	SC	0.2	32.0 ± 9.34	3.86 ± 1.77	391 ± 110	NR	[15]
Alpacas (*Vicugna pacos*)	SC	0.2	6.05 ± 5.34	3.83 ± 2.48	NR	6.20 ± 4.90	[16]
Rabbits (*Oryctolagus cuniculus*)	SC	0.3	34.14 ± 5.47	3.10 ± 1.24	258.40 ± 62.05	4.48 ± 1.36	[18]
Crossbred horse	IM	0.2	33.3 ± 10.5	2.9 ± 0.9	393.6 ± 66.6	9.3 ± 4.3	[20]

Abbreviations: C_max_ = maximum plasma concentration; T_max_ = time to maximum plasma concentration; AUC_0–last_ = area under the plasma concentration–time curve from time 0 to the last quantifiable concentration; AUC_0–∞_ = area under the plasma concentration–time curve from time 0 to infinity; T_1/2λz_ = terminal elimination half-life; NR = not reported. For the present study, arithmetic mean ± SD values were calculated from individual animal data to facilitate comparison with published pharmacokinetic studies. Values for other studies are presented as reported in the respective publications.

## Data Availability

The authors confirm that all data supporting the findings of this research are included within the article.
